# Towards a differentiated understanding of active travel behaviour: Using social theory to explore everyday commuting

**DOI:** 10.1016/j.socscimed.2012.01.038

**Published:** 2012-07

**Authors:** C. Guell, J. Panter, N.R. Jones, D. Ogilvie

**Affiliations:** a Medical Research Council Epidemiology Unit and UKCRC Centre for Diet and Activity Research (CEDAR), Box 296, Institute of Public Health, Forvie Site, Robinson Way, Cambridge, UK; b School of Environmental Sciences, University of East Anglia, Norwich, UK

**Keywords:** UK, Active travel, Commuting, Qualitative research, Social practice

## Abstract

Fostering physical activity is an established public health priority for the primary prevention of a variety of chronic diseases. One promising population approach is to seek to embed physical activity in everyday lives by promoting walking and cycling to and from work (‘active commuting’) as an alternative to driving. Predominantly quantitative epidemiological studies have investigated travel behaviours, their determinants and how they may be changed towards more active choices. This study aimed to depart from narrow behavioural approaches to travel and investigate the social context of commuting with qualitative social research methods. Within a social practice theory framework, we explored how people describe their commuting experiences and make commuting decisions, and how travel behaviour is embedded in and shaped by commuters' complex social worlds. Forty-nine semi-structured interviews and eighteen photo-elicitation interviews with accompanying field notes were conducted with a subset of the Commuting and Health in Cambridge study cohort, based in the UK. The findings are discussed in terms of three particularly pertinent facets of the commuting experience. Firstly, choice and decisions are shaped by the constantly changing and fluid nature of commuters' social worlds. Secondly, participants express ambiguities in relation to their reasoning, ambitions and identities as commuters. Finally, commuting needs to be understood as an embodied and emotional practice. With this in mind, we suggest that everyday decision-making in commuting requires the tactical negotiation of these complexities. This study can help to explain the limitations of more quantitative and static models and frameworks in predicting travel behaviour and identify future research directions.

## Introduction

### Active travel and health

A physically active lifestyle can significantly reduce the risk of obesity and a variety of chronic diseases including coronary heart disease and type 2 diabetes (Department of Health, 2004). However, most adults in the UK do not meet the recommended levels of 30 min of moderate physical activity on at least five days of the week (Department of Health, 2005). Some evidence suggests that engagement in particular types of moderate intensity activities, such as walking and cycling, may be associated with positive health outcomes: for example, adults who regularly walk or cycle to work have higher levels of cardio-respiratory fitness than those who do not (Hamer & Chida, 2008) and commuter cyclists have a lower mortality risk than non-cycling commuters, independent of overall physical activity levels (Andersen, Schnohr, Schroll, & Hein, 2000). Promoting active commuting represents a population approach to increasing physical activity levels; by encouraging everyone to be a bit more active, it may be possible to improve health outcomes across a whole population rather than targeting specific risk groups (Tannahill, 2000). Active commuting may also be an easy way to integrate physical activity into everyday routines, does not require membership fees, and can thus appeal to those less inclined to participate only in more vigorous or structured activities.

That said, many people seem to prefer driving over active travel. While Cambridge, the setting of this study, is one of the few British ‘cycling cities’ (where 18% of trips are undertaken by bike compared to the national average of 2%), cycling is considerably less common in Cambridge than in other European countries such as the Netherlands (Department for Transport, 2010). Research aims to understand active travel as a behaviour that can be predicted and ideally encouraged in interventions. However, evidence from the relatively small number of controlled studies which have explored the impact of interventions on cycling behaviour suggests that their effects have generally been modest and of uncertain statistical significance (Yang, Sahlqvist, McMinn, Griffin, & Ogilvie, 2010). Interventions to promote walking have a somewhat stronger evidence base, although their effectiveness may depend on being targeted on specific groups or settings (Ogilvie et al., 2007).

### Predicting travel behaviour

Research on active commuting is undertaken in several disciplinary fields operating under a variety of assumptions, which may help to explain why interventions have often been found to have only modest success (Killoran, Doyle, Waller, Wohlgemuth, & Crombie, 2006). Some transport research conceptualises commuting within a framework of rational choice (see Metz, 2008), whereby the commuter makes decisions on the basis of minimising the time and other costs of travel. This framework also informs the psychological theory of planned behaviour (TPB) (Ajzen, 1991), which assumes that people hold subjective norms, attitudes and intentions towards certain behaviours and has been used to explain both the ‘unhealthy behaviour’ of car driving and the healthier lifestyle choices of walking or cycling (Forward, 2004), or at least the intention to take them up (Scott, Eves, French, & Hoppe, 2007). Increasingly, these assumptions are problematised in studies which find that people use their travel time usefully for work or leisure (Jain & Lyons, 2008), or which explore the enjoyment of travel and movement (Anable & Gatersleben, 2005). However, these studies have concentrated on motor vehicle use and have not explored the possibility that ‘useful’ travel time could also comprise accumulating physical activity, or enjoying exercise in outdoor surroundings.

A complementary psychological concept to the TPB is that of habit. It assumes that instead of consciously planning journeys, the commuter has developed a routine, for example habitually choosing to use the car parked outside the house every day without reflection. Some studies have shown that these automated and thus unconscious habits override any decision-making or choice and influence travel behaviour over and above attitudes or intentions (de Bruijn, Kremers, Singh, van den Putte, & van Mechelen, 2009), whilst others caution that only high ‘habit strength’ may have this effect (Verplanken, Aarts, & Van Knippenberg, 1997), and that personal and social norms remain important (Klöckner & Matthies, 2004).

Research in transport studies, epidemiology and geography has investigated how individual characteristics, attitudes and beliefs, and social and built environments shape travel decisions. Short distances between origins and destinations are consistently associated with walking and cycling; land use mix and density have been shown to be associated with walking for transport; and the accessibility and safety of neighbourhoods may also be important contributory influences, although the evidence for these is less conclusive (Panter & Jones, 2010; Saelens & Handy, 2008). Moreover, travel behaviour may be influenced by the interplay of infrastructure, neighbourhood characteristics and social circumstances (Pont, Ziviani, Wadley, Bennett, & Abbott, 2009), for example through social support (e.g. that of parents who encourage their children to cycle) or socio-economic status (e.g. while poorer people may live in urban areas of higher density, those environments may also be perceived as threatening for pedestrians in other ways; Bostock, 2001). However, it is often conceded that existing quantitative models can offer only partial explanations of the behaviours of interest (Panter & Jones, 2010): while they suggest that a complex web of physical, psychological, environmental and social factors influence commuting decisions and choices, such complexity is arguably difficult to integrate in a model that aims to simplify and generalise ‘universal’ behaviour.

### Theorising active travel as social practice

Complexity is integral to many social theories that set out to understand the social world and the practices and relationships that shape it. Social theory suggests that “local social organization and the conduct of everyday life are complex, in that they are enacted through multiple modes of social action and representation” (Atkinson, Delamont, & Housley, 2008:31). Bourdieu (1977) argues that people do not act according to rational choice but operate according to a personal set of cognitive and somatic (embodied) dispositions. Bourdieu (1980) calls these dispositions habitus (rather than habit). Habitus is not simplistically understood as automated behaviours but as being driven by the sum of a person's economic capital, social capital (resources based on group membership, relationships, networks of influence and support), cultural capital (e.g. competencies, skills and qualifications) and symbolic capital (e.g. prestige and honour). Bourdieu's habitus thus bridges social structure and individual action. De Certeau's (1984) theory of the practice of everyday life highlights more forcefully that societal and institutional constraints shape how we live. That said, he argues that everyday lives are complex and changeable – for example, social interactions are based on both long-term established relationships and incidental encounters – and that ordinary people use ‘tactics’ to make their lives workable and therefore habitable. Furthermore, his theory of social practice addresses how these tactics include our physical environments and suggests that ordinary actors can actively shape and create meaning for these spaces while moving through them (De Certeau, 1984).

Following such theoretical assumptions, anthropological and sociological accounts are emerging of walking and cycling as social practice. Walking, for example, is not conceptualised as a singular behaviour but as a diverse “everyday practice of social life” (Ingold & Vergunst, 2006:67) and is understood as “an inherently sociable engagement between self and environment” (Ingold & Vergunst, 2006:69). Green's (2009) sociological review of walking suggests that it is a bodily, social and political practice, inherently linked not only to spaces but also to ethnicity and class. Walking can be both a practice of the poor without access to other forms of transport, and a middle-class practice centred around concerns for health, aesthetics and the environment. Cycling, as an intrinsically political action in the context of the ‘cycling-unfriendly’ UK, is often conceived of as an identity-forming practice (Aldred, 2010), while ‘ordinary’ mobility and urban infrastructures, more generally, are re-conceptualised as “sites of (potential) meaningful interaction, pleasure, and cultural production” (Jensen, 2009).

This study aims to contribute to this growing body of theoretical and empirical work on travel and mobility. Rather than focussing on a particular mode of transport it examines commuting as a complex social phenomenon and practice. The qualitative research presented here is part of a wider longitudinal study of travel behaviour and physical activity, the Commuting and Health in Cambridge study (Ogilvie et al., 2010). The qualitative component of this study specifically aimed to explore how people experience and describe travel in their own terms. The analysis provides a rare social anthropological perspective on active travel that focuses on practices of travel and travel decision-making and aims to acknowledge the complexity of social worlds in which these practices have to be negotiated.

## Methods

The research design was conceptualised as an ethnographic approach, gathering varied data with a range of qualitative social research methods in order to arrive analytically at a ‘thick description’ of individual commuting experiences (Geertz, 1973).

### Setting and sample

The setting of this study is Cambridge (population 109,000) and its surrounding hinterland in the east of England, UK (population 494,000 in study catchment area: Census, 2001). Unlike in many other UK cities, cycling is a common mode of travel – not least due to the substantial student population – giving Cambridge the reputation of sporting its own ‘cycling culture’ (Aldred, 2010). However, housing costs in this city, which is within easy commuting distance from London, lead many Cambridge workers to live a considerable distance outside the city, in towns and villages some of which have few public transport options. Many of the participants can make use of the five park-and-ride facilities which surround the city, but often cannot escape the congestion which is present on the main roads around Cambridge.

Of 1164 participants who took part in the baseline survey administered to the Commuting and Health in Cambridge cohort, a sample of those who agreed to be contacted for additional studies were approached for the semi-structured interviews; 49 out of the 74 participants contacted agreed to take part. Participants were purposively sampled according to their gender, home location and mode of transport to explore different social contexts and travel choices in selected neighbourhoods. All 30 participants interviewed by CG were further invited for additional photo-elicitation interviews and 18 participants (plus one partner and three children) agreed to take photos and be interviewed again. The overall sample therefore comprised 21 men and 29 women (plus one boy and two girls) between the ages of 5 and 69, single and with partners, 11 with children under 5 and 12 participants with children between 5 and 15, and some caring for older people. This represents a slightly more diverse sample than the overall cohort sample, who were predominantly women, educated to a degree level (72%) and living in households without children (80%), but nonetheless reflect the largely middle-class white English population of Cambridge. Most of the 50 participants reported using multiple transport modes for commuting, ticking ‘usually’ for various combinations of car, bike or walking in the baseline survey; 11 commuters usually only drove, seven only cycled, and one only walked to work. None reported ‘always’ using any particular mode of transport.

### Research design

Semi-structured interviews were conducted in two phases by two different researchers. NJ conducted 19 interviews between June and August 2009, followed by 30 interviews and 18 photo-elicitation interviews conducted by CG between March and September 2010. Both researchers followed the same interview guide that was focused on travel to and from work, inquiring about typical journeys, routes, modes of transport, time and other factors (such as the need to take children to school) shaping their commuting choices and possible alternatives. A preliminary review and analysis of the first 19 interviews and their transcripts by NJ and CG served as a pilot for the subsequent interviews.

The initial analysis suggested that it would be useful to incorporate another less structured and more participant-driven interview element – photo-elicitation – in the study. For these interviews, participants took photos of one or several commuting journeys, using either their own digital or mobile phone cameras or disposable cameras provided by the research team. Participants were given minimal direction, being asked simply to take some time on their way to or from work to pause and reflect on their commutes. They could document their actual commuting journeys and aspects or experiences of the journeys that were significant to them or an ‘ideal’ (desired) or ‘best day’ commute. Photo-elicitation is a widely used research tool in qualitative health research (Hurworth, Clark, Martin, & Thomsen, 2005) to facilitate in-depth interviewing. The photo-elicitation interview was purely driven by the discussion of the photos.

All interviews were conducted at a place of the participants' choice, at their homes or workplaces or at the research centre, and lasted between 20 and 60 min. They were audio-recorded and transcribed verbatim and the transcripts were double-checked by the researchers. All photo participants were given the opportunity to verify a summary of their initial semi-structured interview. All photos were digitised, and the participants reviewed and selected the photos to be discussed and were given copies of these.

Interviews and photo-elicitation interviews were complemented by detailed ethnographic field notes written by the researchers, during and immediately after each interview. They documented the context in which interview data were produced, recorded other informal interactions, and gathered data on the environmental and social surroundings of participants' homes and journeys. Ethnographic data collection also included general observation of travel practices in the local area and local public discourses around travelling, in order to further contextualise participants' representations of travel behaviour and social and environmental factors (Atkinson et al., 2008).

### Analysis

We analysed a variety of data, namely the interview and photo-elicitation interview transcripts, the participant-produced photos and the field notes. Some participants provided written ‘memos’ with their photos which were also analysed. Unlike other qualitative analytical approaches that focus on content analysis, coding and indexing of textual data, we adopted a more integrative approach to the diverse data set by undertaking ethnographic analysis throughout the data collection process, increasingly ‘funnelling’ the research towards three emerging facets of participants' oral and visual narratives (Silverman, 2006). The analysis was driven by CG as the main researcher, who, for the large part, collected and collated the variety of data. The analysis was peer-checked by NJ to account for the importance of the contextual insights of the researcher collecting the data. Theoretical assumptions and analytical steps were discussed and further developed with JP and DO. Management of the variety of data was aided by the use of NVivo 8.

### Ethics

The study was approved by the Hertfordshire Research Ethics Committee, and all participants gave their written informed consent to the interview study and the photo project respectively. For the photo project, participants received written and oral instructions (including an explanation of the responsibilities of taking photographs in public places), retained copyright of their photos, and gave separate consent to the use of photos for academic dissemination.

## Findings

Three facets of commuting as a social practice and phenomenon emerged from the ethnographic analysis. First, the participants' depictions described the fluid and changing nature of their experiences and life worlds. Second, many participants shared seemingly paradoxical accounts of travel behaviour, e.g. accounts of planning for shortest distances combined with descriptions of the importance of pleasantness of routes. Third, their photographic images produced phenomenological accounts of ‘experiencing commuting’ involving negative but also markedly positive emotions.

### Changing and changeable commuting lives

Each interview started with the question of participants' ‘typical commutes’. Our inquiry was often met with a thoughtful “*it depends*”. Most participants experienced a host of changes in their everyday lives that directly influenced how travel decisions were considered, negotiated and altered. Some of these changes were actively introduced by participants, for example as a means of losing weight or avoiding the unpleasant experience of traffic congestion, but many travel decisions were shaped by their social worlds.

#### Life events

A young woman recounted how she used to commute by bike. “*Before I had [my son], I would cycle every day because it's much easier.*” [aged 34, commutes to work by car or bike]. Depending on her childcare arrangements and her work schedule, however, she had to take the car at some times of the week. When asked if she defined herself as a habitual cyclist or driver she confessed that she quite enjoyed the convenience of driving, especially with her son, and that she “*had to make that conscious decision that I've got to stop using the car now, because I just got in the habit of using the car.*”

Another mother explained that she and her partner were very keen cyclists and used to cycle the journey of over 18 miles together into town some days of the week. The rest of the week she used to take the bus. This changed for her with the birth of their child: “*[O]ur son hadn't started school, our child-minder was too far away to cycle . . . So for that period of time when my son was six months old till . . . school starting, it was really necessary to take the car*” [aged 40, now commutes by bus].

People's lives were constantly subjected not only to major life changes such as moving house, changing job or a child's changing school, but also to less anticipated changes such as the illness of a family member or friend. A woman [aged 62, car] explained: “*I'm a carer for somebody, I’ve had quite a complicated pattern because I was in fact driving . . . to their house first of all to give them their medication and then driving into work.*” Participants' own illness, accidents, or experiences of ageing also affected their commuting choices directly. A keen cyclist [aged 44, car and bike] described the defeat of his own ageing body: “*I just physically can't, my knees can't take it, it becomes painful at that point to do that sort of distance . . . .*”

#### Everyday changes

The participants also experienced smaller changes in their daily lives. Although commuting often reflects everyday routines, these were nonetheless subject to many, more or less unpredictable changes, such as traffic accidents or a car breaking down. As one participant put it: “*It's exceptionally variable. (Laughs) Yesterday, I didn't even get into work because there was a huge lorry fire.*” [woman, aged 39, car and bike]. Commutes varied throughout the year, according to the seasons, weather conditions and traffic congestion that mirrored the school term. It was often those without children who were most acutely aware of these seasonal traffic hotspots. Some people had multiple work sites that required different travel modes; others worked shifts, or had different work schedules on different days.

Families had different arrangements depending on the parents' work schedules and children's school and nursery hours. “*I have to take my son to nursery . . . so I drop him off first. . . . I tend to cycle if I'm going from home to work. . . . But I do work out of the office quite often . . . so yesterday I drove. . . . So it's a bit complicated*” [woman, aged 34, car or bike].

These changes could also be initiated by other people. An academic [aged 68, commutes by car and on foot] explained how his recent change in commuting mode was mostly his wife's idea: “*I cycled from there into town and I did that for two years until . . . I fell off my bicycle in June. . . . I hurt myself quite badly then and now my wife won't let me cycle in town, she says it's too dangerous, which I can't disagree with.*” As changes were experienced within social relationships, they were also negotiated within these relationships.

Changes are anticipated in the travel research literature, in particular in models of ‘habit’ which acknowledge life changes as important opportunities for ‘breaking’ habits and introducing healthier choices (Verplanken, Walker, Davis, & Jurasek, 2008). Two points seem pertinent here: first, many of the changes narrated here were changes towards the car: becoming a carer or having to take children to school or nursery close to the time when the parent's own work day starts. This raises the question of whether major life events or other social changes necessarily provide good opportunities for taking up active travel as some studies suggest (cf. Ryley, 2006). Second, people are, in any case, constantly subjected to small changes, and any travel decisions and subsequent habitual practices are constantly revised and renegotiated (Vergunst, 2008).

### Everyday ambiguous commuting lives

Participants recounted their commutes in shared narratives, evoking common categories of time and space that shape decisions around routes, timings and modes. Nonetheless no two stories were the same, and understandings and explanations varied greatly. Time, for example, was wasted, used, enjoyed or minimised, depending on particular choices and the reasoning behind them, which varied both between and within participants. We suggest that commuting is marked by numerous ambiguities in reasoning, ambitions and roles.

#### Ambiguous reasoning

During her interview a woman [aged 39] gave a passionate account of her choice to commute to work by bus. She cherished the spare time she gained through her bus travel, chatting to her “*bus buddies*”, reading novels or watching the world go by and the changing scenery throughout the year. Most other participants did not consider the local buses a viable option for their daily commute; they felt bus travel to be a waste of time, objecting to the delays and the long routes taken through every village on the way. This woman, however, saw it differently: “*Occasionally it's late but I've given up worrying and fretting about being late in to work, I think that's what you've got to get over, . . . if the bus breaks down . . . what can I do about it, you know, wait for the next one.*”

She seemed not to share the public script of limiting commuting time and searching for the quickest option possible; as several other participants put it, commuting “*eats into the day*”. This woman's travel choice seemed to reflect those studies which find that some people do not regard travel time as wasted (Jain & Lyons, 2008). Part of her defence of bus travel, however, was her aversion to the traffic she was subjected to as a car driver and she could not understand her colleagues' choice. She used the same argument her colleagues might use against her pleas to use the bus, one of time wasting: “*One of the Sisters at work missed a study day this week . . . because she couldn't get parked. . . . had she got the bus she wouldn't have been waiting in the queue to get in to the car park for nine o'clock.*”

Her reasoning around time and its use here may appear ambiguous or even paradoxical, but she was not an isolated case in this regard. A waste of time could also refer to other commuting choices, for example wasting time in traffic rather than, for example, getting some exercise while cycling. “*Because with a car . . . my journey to work is just a complete waste of time, it's just time that I could be using doing something else*”, reasoned one participant [woman, aged 34, car or bike]. From a social science perspective, these multiple ways of ordering are a common feature of social lives. The social world and the way we make sense of it is complex because “[t]hings add up *and* they don't. They flow in linear time *and* they don't” (Mol & Law, 2002:21; their emphasis).

#### Ambiguous aspirations

A man [aged 61] who cycled to work every day talked about the flexibility and independence of his commuting journeys. Knowing all the corners and alleyways of his home town of 60 years, he relished the daily defeat of the regular traffic jams that stress Cambridge commuters. Part of his photo story, however, was a picture of two old men ushering a flock of ducklings into the river Cam. Looking at this tranquil early morning scene, he explained: “*I remember this poem, you know it's: ‘What is this life if full of care, we have no time to stand and stare’. You know I'll stop on [the bridge] and watch the guy with the ducks, or I'll watch the punts going past*”. His interview echoed that of many cyclists who emphasised the joy of “whizzing past” stationary traffic, but equally considered themselves much more aware of their surroundings, being able to stop at any time to admire the views. Many of the participants who deliberately choose the park-and-ride option to avoid sitting in traffic for unpredictable periods of time described the cycling or walking part of their journey as a welcome ‘time out’, providing time for reflection and a brief pause in their busy schedules.

#### Ambiguous identities

These narratives are all different and feature different ambiguous or paradoxical categories. Quite often, it seemed difficult to ascribe clear commuter roles to participants. A young mother [aged 31] explained: “*Commuting is always the car. Wouldn't be anything else in reality, purely ‘cos of convenience and time I have to be in the office. . . . Nursery opens at 8 and I can't cycle that fast.*” According to her main commuting choice, she would be classified a car commuter. Using the car, however, was not her quickest or first choice – or even habit – in all circumstances. For fetching her son from the nursery, “*it's usually the bike, the bike's quicker. It's . . . not far but it's just an excuse to kind of get them used to [the] bike*”. Her explanations make clear that for this trip the bike was a quicker choice in comparison with, say, trying to find a parking space for that part of the school run. That said, she also wanted her son to experience a cycling lifestyle such as the one with which she had grown up. Pinning her attitudes, intentions or even behaviour down to singular decisions does not adequately reflect her social lifeworld and practices. In fact, many of the participants – not only the ‘multi-modal’ users of park-and-ride facilities – could not be ascribed clear identities as cyclists or car drivers (cf. Anable, 2005), as many of the previous examples have also shown. Depending on the setting, the time of day or the destination, travel choices can change and become more or less viable, desired or habitual.

### Everyday embodied commuting lives

Many of the semi-structured interviews were about very practical concerns: modes of transport, safety, routes and variations, work hours and rush hours. However, the participants' photos and subsequent photo-elicitation interviews highlighted another important aspect of commuting. Commuting as the daily recurring activity of driving a car, sitting on a bus, cycling around potholes, enjoying a favourite radio programme or a scenic bike ride conjured up a range of physical and emotional experiences, both negative and positive, of stress and danger and of wellbeing and enjoyment. Anthropologists ‘of the body’ (Lock, 1993) urge us to consider the actual bodily experience of practices, not merely in what ways we make decisions, rationalise or habitually engage in behaviours. How we feel and sense when engaging in practices shapes our decision-making processes and the meanings we give to these; practices become ‘embodied’ (Lock, 1993:137).

#### Scar(r)ed commuting bodies

As expected, most car commuters shared negative experiences of spending a substantial part of the day sitting in traffic. A participant [woman, aged 62, car] summarised these experiences poignantly: “*I think commuting is bad for your health because it is quite stressful and it takes up a . . . large part of your day.*” This young mother [aged 34] made a direct comparison between her car and cycle commute: “*I find the car journey far more frustrating and irritating . . . whereas on a bike I never feel like that at all, I'm just completely in a relaxed little zone.*” These narratives of stress, frustration and ‘bad moods’ were not only considered ‘bad for people's health’ but also often recounted in an agitated and emotional way. As Green (2009:27) suggests, the activity of walking “is a largely taken-for-granted accomplishment” and this could easily be extended to commuting as an ordinary and frequent practice. It is then the negative and adverse that is noticed, when “resistance (at least in specified times and places) to the instrumental, hurried, convenience of urban environments” is felt (ibid.). Commuting then becomes both an ‘embodied’ and an ‘embedded’ practice in space and time.

For cyclists, these embodied commuting practices were connected to a real sense of danger – both imagined and painfully experienced. Most cyclists could list a range of ‘near-misses’ or actual collisions. A woman [aged 57] who is now mostly driving to work recalled: “*I mean there was one occasion where I was going round a roundabout and a car cut me up on the inside. . . . He put my life at risk basically.*” A regular cyclist [man, aged 62] described these experiences as a shared narrative at Cambridge dinner parties: “*Everybody has experiences, yes, so when they talk about them then you find that all the cyclists will . . . get uppity very quickly because . . . it's fright; it's a series of frightening experiences.*”

The participants connected these dangers to certain spaces. “*There's a little mini-roundabout and I've nearly been knocked off my bike there several times by cars . . . So I tend to get off and be a pedestrian around that if I'm going that way*” [woman, aged 45, bike]. This sense of danger did not only affect those who chose active travel modes, as this woman [aged 38, bus] described the road she used to drive regularly to work: “*Yeah, it's really dangerous. Well that's another reason for not going back to driving my car. There have been so many people killed on that road, it's heartbreaking.*”

#### Sensual commuting bodies

However, participants' descriptions of their commutes also contained many expressions of enjoyment. This was at first a surprising finding, and we suggest that being given the task of documenting these activities may have helped participants to reflect on these more sensual experiences. This young woman [aged 34] enjoyed both the exercise and the scenery of her cycling commute: “*. . . to get some exercise on the bike definitely and with the sun being out last week that was just glorious.*” Another participant [aged 46] who regularly walked from the park-and-ride facility to work commented on her photos: “*Yeah, lovely little buttercups, so it's really very pleasant so it lifts my spirits when I walk along*.” Again, these narratives reflect embodied experiences of their commute; the spirituality of a walk or the uplifting sight of nature.

While many of the cyclists shared images of beautiful green landscapes, or expressed their enjoyment of getting some exercise or being able to wind down after work, other transport choices seemed to produce similar pleasant experiences. This woman [aged 57] had recently moved out of town and become a regular car driver, and although she used to cycle and was a nervous driver at first, she described the positive change in her life, away from cycling ‘near-misses’ towards tranquil car journeys: “*I'm really enjoying it . . . I think having had thirty years of cycling in all weathers it's a really nice change to be able to turn the radio on and catch up with a bit of news and . . . just chill really, and prepare for the day.*” Similarly, this bus user [woman, aged 38] explained: “*I'm sitting upstairs on the bus here and . . . it's absolutely beautiful . . . in the spring there's bluebells and you can see them when you're upstairs . . . and it's just really lovely.*”

These expressions of wellbeing, enjoyment and even happiness stand in stark contrast to the negative tales of accidents, road rage, hazardous cycle lanes and potholes. While these distressing issues might be more vocally and self-evidently raised (Mathews & Izquierdo, 2009), often with a clear and pressing political agenda, the quieter stories of wellbeing teased out through photography show a flip-side to the very immediate way in which we engage with our environment during our daily commutes. Perhaps it is these aspects of wellbeing and joy on which commuters draw in order to make them as resilient as they are, getting back on the bike after accidents because the exercise would be missed, or queuing every day at the same road junction because the chance to listen to the car radio is much preferred to a bumpy ride on a crowded bus.

## Discussion and conclusion: commuting tactics

Our findings illustrate the value of qualitative research informed by social theory in exploring and understanding the practice of commuting, which is deeply embedded in our social lives and reflects the multiplicity and messiness of everyday life (O'Brien, 2009). Social theories aim to acknowledge this complexity without reducing its description to simplistic accounts. “There is complexity if things relate but don't add up, if events occur but not within the process of linear time, and if phenomena share a space but cannot be mapped in terms of a single set of three-dimensional coordinates.” (Mol & Law, 2002:1) While quantitative epidemiological research on travel behaviour aims to understand the influence of a variety of psychological, social, physical and structural factors, it has generally struggled to account for much of the observed variance in behaviour or the interactions between, for example, individual psychology and the physical environment (Panter & Jones, 2010). The very nature of theoretical frameworks and causal models requires complexities and ambiguities to be simplified, rendering fluid social lives into more static, measurable forms. Nonetheless, part of the problem may lie in the tendency to describe commuting simplistically as ‘a behaviour’ – rather than a bundle of beliefs, values and activities – and ascribing labels such as a ‘car commuter’ (cf. Anable, 2005).

The young mother interviewed in this study guiltily described her frequent use of the car as a ‘bad habit’ she hoped to give up, while simultaneously regarding herself (like many ‘born and bred’ Cambridge residents) as a habitual cyclist. This possibility of identifying with more than one habit or identity (‘motorist’, ‘cyclist’, ‘pedestrian’ and so on) at the same time challenges the assumption, implicit in most public discourse about transport policy, that such groups might be discrete and even hostile, as in the notion of a ‘war on the motorist’ (Department for Communities and Local Government, 2011). This participant found a mixture of driving and cycling to be most suitable for her current daily life, which required her to manage her work and family responsibilities in a carefully planned routine, but this had not always been the case. Her uneasy feelings towards driving may have been partly informed by the attitudes and values inculcated by growing up in a cycling ‘culture’ (Aldred, 2010), but planned behaviour as social practice is more than the sum of attitudes, norms and intentions. It may also be unhelpful to regard habit as reducible to a singular automated behaviour that can be assessed by agreement with a list of statements (Verplanken et al., 1997). Bourdieu's (1977) habitus describes ‘habits’ as practices which are informed by the sum of our previous experiences, the socio-cultural background of our social class that determines our values and preferences, social relations and access to financial and intellectual resources. All of these help shape our habitual practices and decisions as well as our understandings and attitudes towards, for example, time and how we use it. If practices are only established because they work well within people's social lives, it follows that a more informed approach to research in this area should also investigate how habits are formed, rather than merely measuring their ‘strength’ (Forward, 2004).

Quantitative research increasingly emphasises the importance of distance in influencing decisions about where to live and work (Panter & Jones, 2010), which contribute to shaping the travel choices of commuters (Rodriguez, Levine, Agrawal, & Song, 2011). Infrequent major life events such as moving house, changing job, or the birth of a child therefore make it almost inevitable that habitual practices such as commuting patterns will be re-evaluated and therefore present an opportunity for shifts in mode choice (Verplanken et al., 2008). However, ‘context changes’ of this kind, and the ‘window of opportunity’ they present, need not be limited to major disruptive events, since organising our social lives also requires the skill to negotiate the constant, more minor changes that arise day by day or week by week. As De Certeau (1984) argues, in everyday life ordinary people are actively engaged in social relationships, the conditions in which they live and work, and the physical environment through which they move. People manoeuvre tactically through these adversities, established relations or haphazard encounters, and in the context of commuting, people are constantly renegotiating the organisation of their daily travel: responding to seasonal, financial, familial and emotional changes, creating their own spaces, rendering them dangerous or safe, stressful or even enjoyable.

Finally, we argue that participants in this study did not engage in social practices as individual agents. On the contrary, many decisions were made and choices offered or denied within the social contexts of family, work or local infrastructures. A man changed his mode of transport because his accident frightened his wife; a mother changed her commuting routines because her childcare arrangements clashed with her work schedule; an apprehensive woman bought a car and became an avid driver. These aspects of the social context form part of the explanation for the travel choices made and cannot simply be treated as ‘confounders’ in the analysis of other explanatory factors. Similarly, the travel choices made by individuals in moving through a shared transport network help to shape the context in which others make their decisions, as cyclists engage in actions of resilience or defeat in response to dangerous interactions with other traffic participants.

The limitations of the study should be acknowledged. The study focused on the very particular setting of Cambridge and drew on a sample of highly educated and eloquent participants, and the analysis was thematically guided by the theoretical position and interest of the first author. Nonetheless, as with most qualitative social research the aim of this work was to produce in-depth, exploratory and explanatory findings rather than to address expectations of representativeness or generalisability. There is an increasing aspiration to capture complexity in evaluative frameworks such as those for ‘complex interventions’ (Craig et al., 2008), and therefore an expectation that the social sciences might provide more contextualised data with which to do this. One potentially valuable contribution demonstrated in this study would be a more differentiated understanding of ‘behaviours’ identified as risk factors for ill-health (Armstrong, 2009) as social complex phenomena that require to be ‘unpacked’.

It remains an intriguing challenge to explore how research of this kind that acknowledges the complexity of social worlds can most usefully be translated and applied to inform those more systematic frameworks. More qualitative research would help to strengthen a more complex, integrative approach. This study has shown the value of further exploring the influence of social and collective interaction, focussing more on households and joint decision-making rather than simply concentrating on individuals, and investigating wellbeing and enjoyment as they relate to travel. Future research should acknowledge that preferences, vested interests and choices may vary depending on social roles or chores or between population groups (e.g. see Walker, 2011) and should therefore depart from a narrow focus on travel mode, instead teasing out such social obligations and their connections to choice and opportunity. This could directly inform practice by helping those delivering interventions to tailor their approach to the diverse social contests of their target populations.
